# Transcriptional remodeling of cardiomyocytes and fibroblasts during post-myocardial infarction recovery

**DOI:** 10.1038/s41598-026-41631-y

**Published:** 2026-03-04

**Authors:** Pankaj Singh Dholaniya, Helena Islam, Syed Baseeruddin Alvi, Muhamad Mergaye, Onur Kanisicak, Mahmood Khan

**Affiliations:** 1 Division of Basic and Translational Research, Department of Emergency Medicine, College of Medicine, The Ohio State University, Columbus, OH 43210 USA; 2 Department of Physiology and Cell Biology, The Ohio State University, Columbus, OH USA; 3 Davis Heart and Lung Research Institute, The Ohio State University, Columbus, OH USA; 4 Department of Biotechnology & Bioinformatics, School of Life Sciences, University of Hyderabad, Hyderabad, India

**Keywords:** Cardiology, Cell biology

## Abstract

Myocardial infarction (MI) results from reduced coronary blood flow, leading to oxygen deprivation and impaired systolic and diastolic function, which increases the risk of cardiac arrhythmias. Various cardiac cell types respond to this stress to preserve heart function, but the precise, cell-type-specific mechanisms remain poorly understood. To investigate these responses, we performed single-nucleus RNA sequencing (snRNA-seq) on left ventricular tissue from mouse hearts at baseline (Day 0) and at 1 and 4 weeks post-MI. This enabled us to characterize transcriptional changes across major cardiac cell types. We observed significant shifts in the transcriptional states of cardiomyocytes (CMs) and fibroblasts (FBs) cell populations following MI. CMs showed a major transcriptional modulation from healthy to diseased state during early chronic phase of post-MI, however, the recovery phenotype was observed during the late chronic phase, suggesting a natural compensatory response of CMs against the ischemic stress. FBs exhibited dynamic transcriptional changes consistent with roles in post-MI healing and fibrosis. In addition, inferred alterations in cell-cell communication networks highlighted changes in intercellular signaling pathways, shedding light on disrupted crosstalk in the injured heart. Together, our findings provide a comprehensive transcriptional landscape of cardiac cell populations, especially CMs and FBs, following MI and identify potential molecular targets for therapeutic intervention.

**Supplementary Information:**

The online version contains supplementary material available at 10.1038/s41598-026-41631-y.

## Introduction

Myocardial infarction (MI), commonly known as a heart attack, is a critical medical condition characterized by the obstruction of blood flow to the heart muscle, leading to tissue damage^1–3^. It is primarily caused by atherosclerotic rupture, leading to the abrupt occlusion of a coronary artery, which deprives the heart muscle of oxygen and essential nutrients^1,4^. The impact of MI on the heart tissue is very profound which leads to irreversible damage to the heart tissue, leading to cell death and scar formation. After MI the functional cardiomyocytes are lost and replaced by scar tissue, compromising the overall functional efficiency of the heart^5,6^. ​​This structural remodeling significantly impairs the contractile function of the heart, increasing the risk of ventricular dysfunction, arrhythmias, and heart failure in the long term. The extent of damage and the subsequent repair processes vary depending on factors such as the severity of ischemia, the efficiency of revascularization, and the activation of molecular pathways involved in cell survival, inflammation, and fibrosis^7–9^.

At the molecular level, MI induces widespread transcriptomic changes across various cardiac cell types, primarily affecting cardiomyocytes (CMs), endothelial cells (ECs), cardiac fibroblasts (FBs), and immune cells. The major transcriptional changes include the upregulation of immune-related genes, cell-cycle-related pathways, and extracellular matrix remodeling genes^6^. Notably, MI significantly impacts energy metabolism in CMs, shifting their primary energy source from oxidative phosphorylation and fatty acid oxidation to glycolysis as an adaptive response to ischemia^10–13^. Although this shift from fatty acid oxidation to glycolysis is not energy efficient, it serves as a temporary survival mechanism, enabling CMs to sustain ATP production in an oxygen-deprived environment^14–16^.

Following MI, CMs also undergo morphological and structural changes that collectively contribute to cardiac remodeling and dysfunction^17^. Ischemic CMs exhibit cellular swelling, loss of intracellular organelles, and disruption of myofibril architecture resulting in the death of CMs either via necrosis or apoptosis^18–20^. In surviving CMs, hypertrophy, an increase in cell size, is a prominent feature, driven by the need to compensate for the loss of contractile function in the infarcted region^21,22^. However, this compensatory hypertrophy is often maladaptive, leading to increased wall stress, impaired contractility, and consequently, heart failure. Furthermore, CMs after MI undergo cytoskeletal remodeling, characterized by alterations in the expression and organization of proteins such as actin and desmin, which are essential for maintaining cellular structure and mechanical integrity^23,24^. In addition to this, changes in the extracellular matrix, including increased collagen deposition and crosslinking, lead to myocardial stiffening and reduced compliance, further impairing cardiac function^25^. The week 1 (Wk-1) post-MI marks a crucial transition phase where the heart shifts from acute inflammation to early repair mechanisms. Within the Wk-1 post-MI, the immune responses begin to resolve, allowing for the activation of FBs and the initiation of extracellular matrix remodeling^4,26^.

FBs are the key responder to ischemic stress^27,28^. The major post-MI cellular changes in different cardiac cells are centralized to FB signaling^29,30^. Immediately after MI on day 1 FBs activate the inflammatory signaling followed by proliferative and pro-angiogenic signaling which peaks at day 3. Later the angiogenic signaling reduces and FBs promote scar formation by secreting extracellular matrix (ECM) at day 7^31^. By week 4 (Wk-4), the heart enters the chronic remodeling phase, characterized by mature scar tissue formation and ongoing fibrosis, which can significantly impact cardiac function^32,33^. Studying these time points may provide valuable insights into the temporal dynamics of cellular and molecular changes, highlighting potential therapeutic targets for improving healing and limiting adverse remodeling in the post-MI heart.

Understanding the post-MI cellular dynamics using single-cell/nuclei RNA sequencing (sc/snRNA-Seq) has gained increasing attention in recent years^34,35^. This allows for identifying the post-MI gene expression changes at single-cell level, along with associated molecular pathways and cell-cell communication, which is crucial for developing novel therapeutic strategies. Along with the functional modulations in cardiac cells, the distribution of cell number is also reported to be changed post-MI, this includes a reduction in CMs and ECs along with an increase in immune/inflammatory cells and FBs^36^. In this study, we performed the single-nuclei RNA sequencing (snRNA-Seq) of the left ventricle region isolated from the mouse heart post-MI at Wk-1 and Wk-4, along with a control heart sample. We specifically looked at the remodeling process that takes place in surviving CMs at Wk-1 and the long-term chronic phase characterized by mature scar tissue formation at Wk-4. Our study highlights the key modulations, specifically in CMs at the gene expression level, that explains the transcriptional modifications after cardiac ischemia. Further, we investigated the intercell communication to highlight the major signaling pathways between FBs and CMs that could be involved survival of CMs post-MI.

## Results

### MI via permanent LAD ligation shows an overall reduction in cardiac function at Wk-1 and Wk-4

**Fig. 1:**
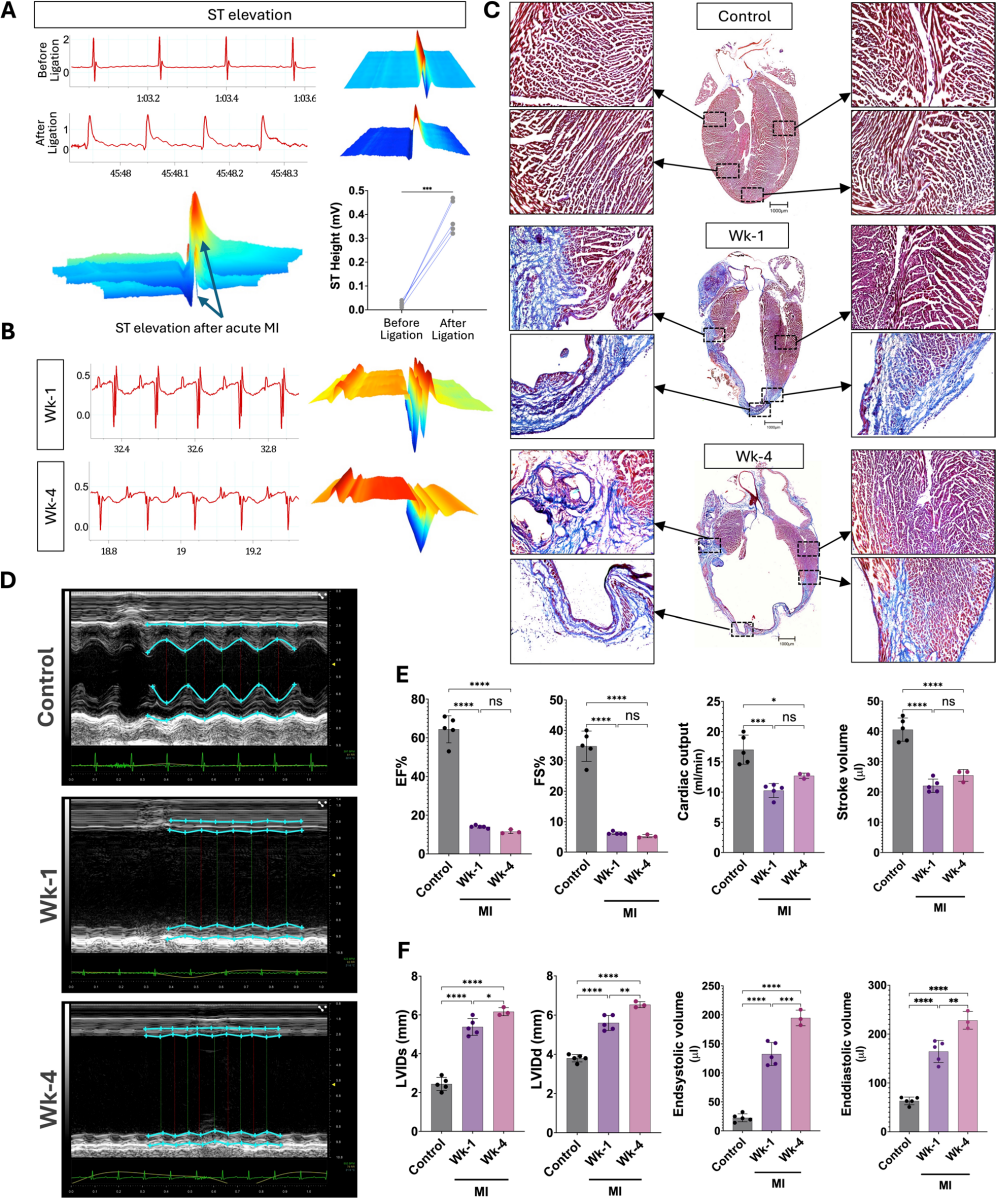
Alteration in surface electrogram and Impairment of Left ventricular function at Wk-1 and Wk-4 Post-MI mice. **A**  Electrocardiogram showing the ST elevation after ligation demonstrated by ECG traces and waterfall plots. **B** The ECG traces and waterfall plots at post-MI Wk-1 and Wk-4. **C** Masson’s trichrome (MT) staining of longitudinal sections at the point of ligation of the left ventricle from Control, Wk-1, and Wk-4 post-MI mice hearts. **D** Echocardiogram recording from Control, Wk-1, and Wk-4 post-MI mouse. **E**, **F** Changes in echocardiographic parameters during post-MI Wk-1 and Wk-4. Statistical significance of the difference between control and WK-1 & WK-4 of MI mice was assessed by one-way ANOVA test (Tukey’s multiple comparison); **p < 0.01 and ****p < 0.0001).

All the mice subjected to LAD ligation showed a significant decline in cardiac function. The ECG showed clear ST-segment elevation immediately after LAD ligation (Fig. 1A), with distinct alterations in ECG morphology observed at both Wk-1 and Wk-4 post-MI (Fig. 1B). The MT staining demonstrates the scar region (blue color) in the left ventricle of the heart at Wk-1 and Wk-4. The LV wall thinning was very evident resulting in dilation of the heart (Fig. 1C). This is further confirmed by the echocardiography which clearly shows the wall thinning and the decrease in LV function (Fig. 1D). The functional parameters such as EF, FS, cardiac output, and stroke volume were significantly decreased post-MI, however, there was no significant change observed between Wk-1 and Wk-4. (Fig. 1E). The LVIDs, LVIDd, end-systolic and end-diastolic volume were gradually increased from control to Wk-4 post-MI (Fig. 1F).

### MI leads to compositional changes in cell type in the heart

**Fig. 2:**
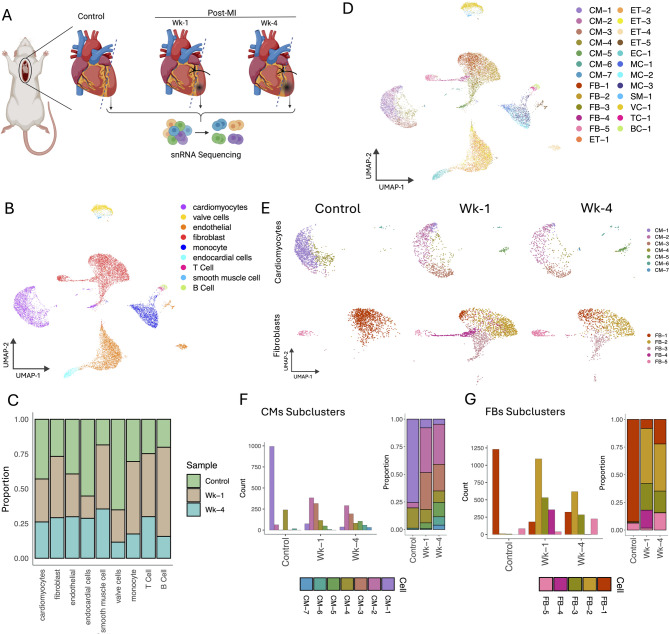
Compositional changes in cardiac cells post-MI. **A** Schematic representation of experimental plan showing the collection of the left ventricle from the control mice heart and post-MI Wk-1 & Wk-4 subjected to snRNA sequencing. **B** The UMAP plot showing 9 annotated cell clusters. **C** The proportional distribution of the number of cells across samples for each cell type. **D** Distribution of subclusters for each annotated cell type. **E** UMAP plots demonstrate the change in cluster composition for cardiomyocytes and fibroblasts across different samples. **F**, **G** The distribution (counts and proportions) of cells in subclusters across different samples for cardiomyocytes and fibroblasts. CM: Cardiomyocytes; FB: Fibroblasts; ET: Endothelial cells; EC: Endocardial cells; SM: Smooth Muscle cells; VC: Valve cells; TC: T-Cells; and BC: B-Cells.

The unsupervised clustering of snRNA-Seq data from Control, Wk-1, and Wk-4 post-MI and cell annotation analysis identified major clusters with 9 different cell types (Fig. 2A, B). The number of CMs was observed to decrease post-MI, potentially demonstrating loss of CMs, whereas the number of FB was initially increased at Wk-1, suggesting a fibrotic response to the ischemic stress (Fig. 2C). These 9 major cell clusters contain a total of 25 transcriptionally distinct subclusters across all samples (Fig. 2D). Cluster heterogeneity for each cell in different samples is shown in Supplementary Fig. 1. Notably, a substantial proportion of these clusters were assigned to CMs (7 clusters) and FBs (5 clusters), together comprising nearly half of all identified clusters. This predominance reflects the transcriptional heterogeneity within these two major cell types, especially in the context of post-infarction remodeling. Given that CMs are the primary contractile units of the heart and FBs are central to extracellular matrix (ECM) remodeling and fibrosis, the diversity observed within these populations likely reflects their dynamic and multifaceted roles during cardiac injury and repair. Consequently, we focused subsequent analysis on characterizing these CMs and FBs subclusters to uncover key transcriptional programs, stress responses, and signaling pathways associated with myocardial infarction and its progression over time. For instance, the cluster “CM-1” within CM populations was the predominant cluster under control conditions, but it was largely displaced by clusters CM-2 and CM-3 in the post-MI Wk-1 and Wk-4 samples, indicating a substantial transcriptional shift in CM populations following MI. Similarly, cluster FB-1, a major cluster in FBs populations under control conditions, was replaced by a significantly larger number of cells in clusters FB-2, FB-3 and FB-4 (Fig. 2E-G).

### Key transcriptional variations and appearance of disease state in cardiomyocytes

**Fig. 3:**
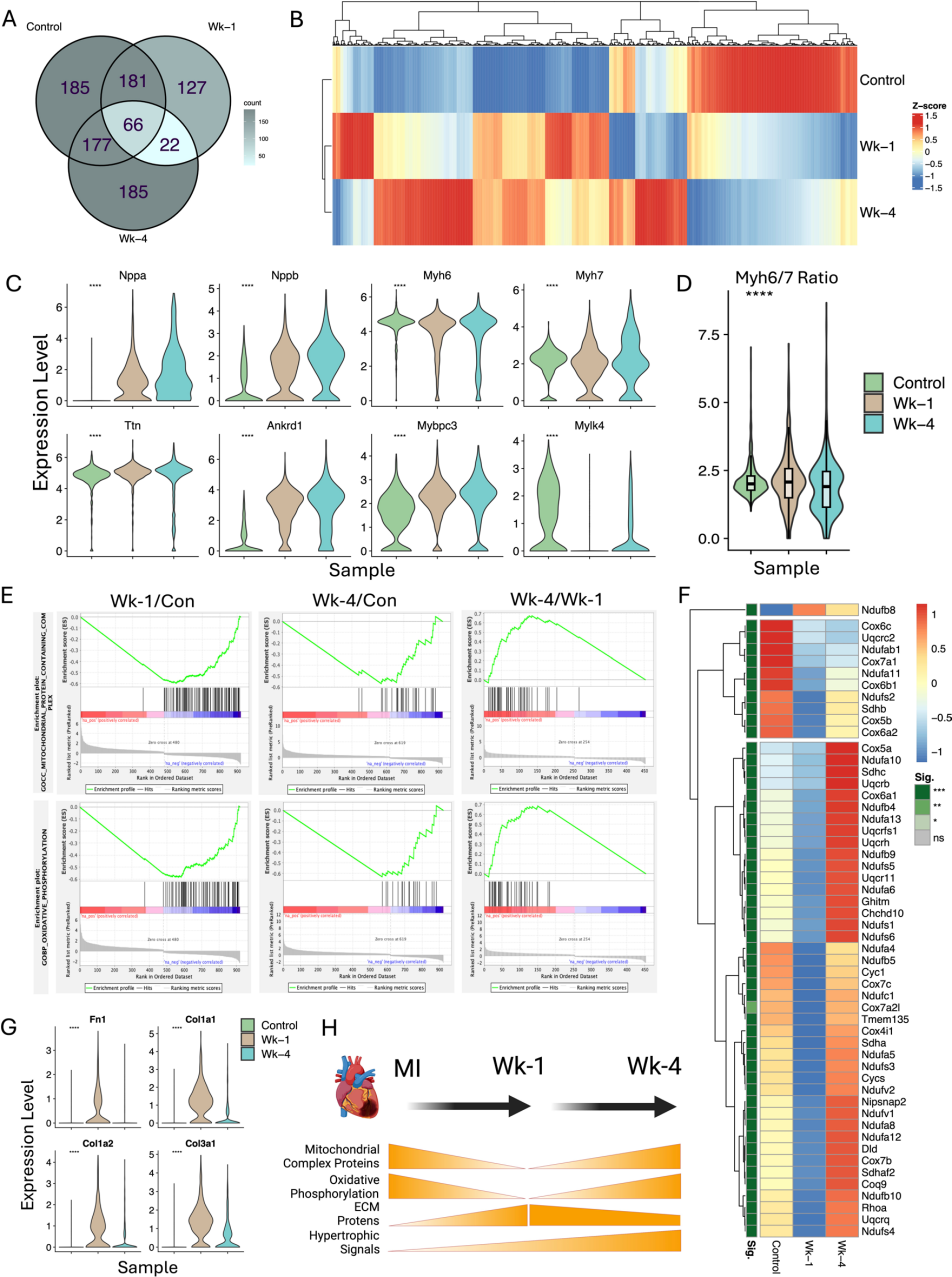
Transcriptional variations during the disease state in CMs post-MI. **A** Venn diagram showing the overlap and distinct sets of differentially expressed genes (DEGs) in CMs across control, Wk-1, and Wk-4 post-MI conditions. **B** Hierarchical clustering heatmap of CM transcriptomes indicating distinct gene expression profiles among control, Wk-1, and Wk-4 samples. **C** Violin plots demonstrating increased expression of Nppa, Nppb, Myh6, Myh7, Ttn, Ankrd1, Mybpc3, and Mylk4 in CM clusters post-MI. **D** Violin plot showing the change in Myh6/7 ratio post-MI. **E** The gene set enrichment analysis (GSEA plots) for DEGs shows a significant enrichment in mitochondrial protein subunits and oxidative phosphorylation pathways. Most genes were downregulated in Wk-1 and then restored their expression by Wk-4. **F** The heatmap shows the expression pattern of mitochondrial subunit & oxidative phosphorylation proteins. **G** The expression of ECM genes (Fn1, Col1a1, Col1a2, Col3a1) was found to increase during Wk-1 post-MI in CMs. **H** The DEGs patterns demonstrate that there is a decline in mitochondrial protein subunits and oxidative phosphorylation genes during Wk-1, which is gradually restored during Wk-4. The expression of hypertrophic-related genes gradually increases by Wk-4.

Differential expression analysis of the CMs cluster revealed substantial transcriptional alterations at both Wk-1 and Wk-4 post-MI **(**Fig. 3A, B**)**. The expression of CMs injury marker genes Nppa and Nppb and several other hypertrophic marker genes such as Ttn, Ankrd1, and Mybpc3 was gradually increased during the Wk-1 and Wk-4 post-MI (Fig. 3C). The expression of Myh7 also increased during Wk-1 and Wk-4 as compared to the control. In contrast, the expression of Myh6 is decreased, resulting in a post-MI shift in the Myh6/Myh7 ratio, which suggests that the surviving CMs exhibit a hypertrophic phenotype (Fig. 3C-D & Supplementary Fig. 2). The WGA staining of the cardiac sections further confirms the increased cell size post-MI Wk-1 and Wk-4 in the peri-infarct region (Supplementary Fig. 3). It has been reported that ischemic stress in CMs leads to a metabolic shift toward increased glucose utilization^12^. Gene Set Enrichment Analysis (GSEA) of differentially expressed (DE) genes across all CM clusters at Wk-1 and Wk-4 reveals downregulation of mitochondrial complex proteins and the oxidative phosphorylation (OxPhos) pathway post-MI (Fig. 3E). However, when comparing Wk-4 to Wk-1, the DEGs show a reversal effect, indicating an increase in OxPhos pathways. This reflects a compensatory response in CMs as a decrease in oxidative phosphorylation during Wk-1 post-MI, followed by an increase by WK-4 (Fig. 3E). We further looked at the expression of genes enriched in these pathways. The results indicate a decreased expression of several subunits for mitochondrial proteins during Wk-1 and their expression level was restored to normal level in Wk-4 post-MI (Fig. 3F). The expression of ECM genes (Fn1, Col1a1, Col1a2, Col3a1) was increased during Wk-1 post-MI followed by a decrease in Wk-4 (Fig. 3G). Overall, these results suggest a temporary decline in the mitochondrial activity and oxidative phosphorylation in CMs at Wk-1 post-MI, followed by an increase during the Wk-4 suggesting a compensatory response in surviving CMs. However, the hypertrophic markers gradually increased during Wk-1 and Wk-4 post-MI (Fig. 3H).

### Stabilization of fibrotic scar formation at Wk-1 post-MI

**Fig. 4:**
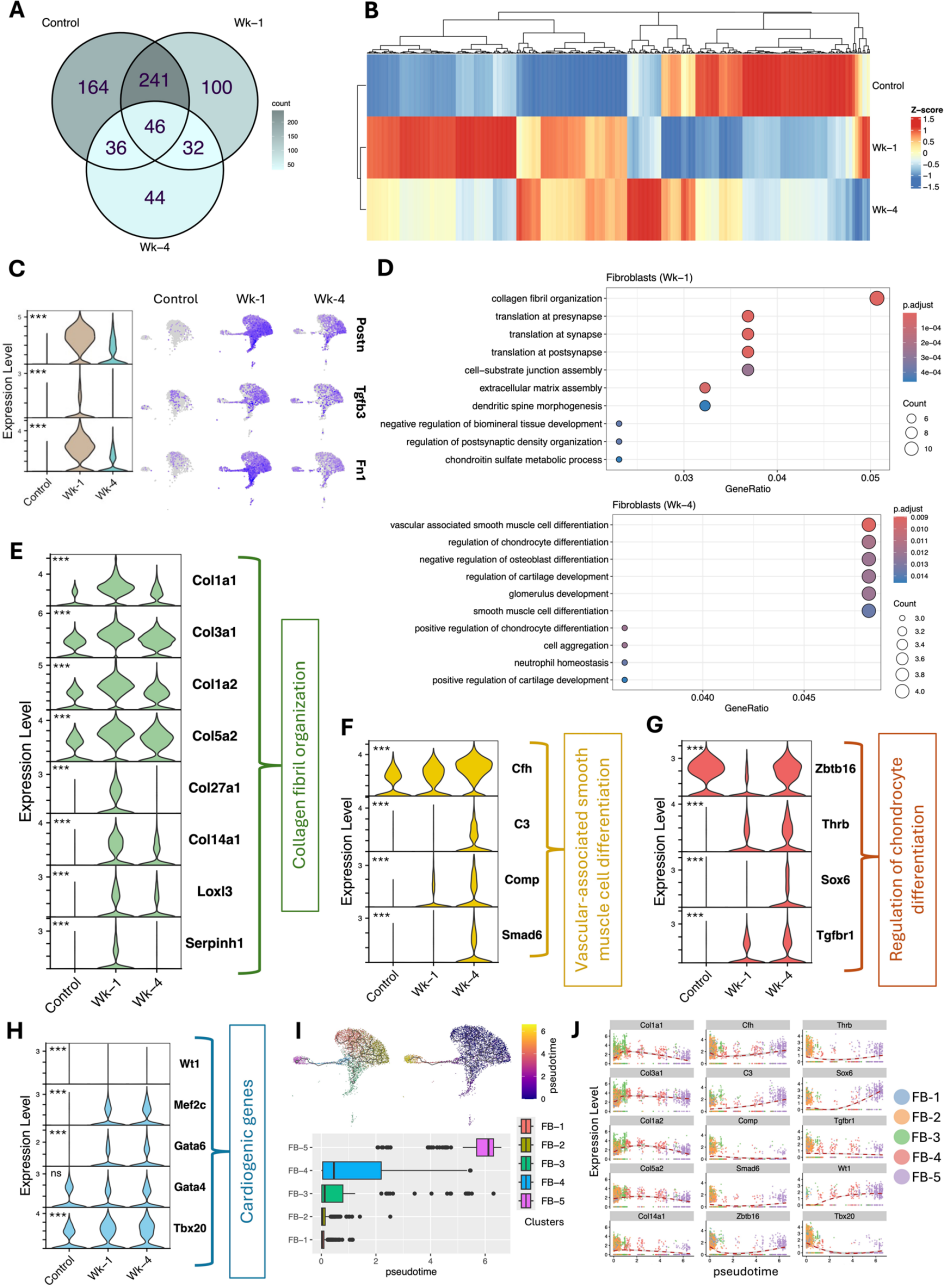
Differential gene expression analysis in FBs. **A** Venn diagram showing the overlap and distinct sets of differentially expressed genes (DEGs) in FBs across control, Wk-1, and Wk-4 post-MI conditions. **B** Hierarchical clustering heatmap of FB transcriptomes indicating distinct gene expression profiles among control, Wk-1, and Wk-4 samples. **C** Violin and feature plots illustrating the expression of key FB activation markers (*Postn*, *Tgfb3*, *Fn1*) across conditions, with highest expression observed at Wk-1 post-MI. **D** GO enrichment analysis of DEGs in FBs at Wk-1 (top) and Wk-4 (bottom). Wk-1 FBs were enriched for extracellular matrix (ECM)-related processes, while Wk-4 FBs showed enrichment for pathways related to vascular and chondrocyte differentiation. **E** Violin plots showing expression levels of genes involved in collagen fibril organization (*Col1a1*,* Col3a1*,* Col1a2*,* Col5a2*,* Col27a1*,* Col14a1*), ECM regulation (*Loxl3*,* Serpinh1*), which are upregulated at Wk-1. **F** Violin plots displaying increased expression of genes associated with vascular-associated smooth muscle cell differentiation (*Cfh*,* C3*,* Comp*,* Smad6*) at Wk-4. **G** Violin plots showing expression of genes involved in regulation of chondrocyte differentiation (*Zbtb16*,* Thrb*,* Sox6*,* Tgfrb1*) in FBs, also elevated at Wk-4. **H** Violin plots showing expression levels of cardiogenic genes and **I** Pseudo-time analysis of FB cluster demonstrating the trajectories identified and the pseudo time across different subclusters. **J** The expression pattern of the genes during pseudo time across different subclusters.

The differential expression analysis of the FBs cluster revealed significant transcriptional changes post-MI at Wk-1 and Wk-4. A Venn diagram of DEGs revealed both shared and time-specific transcriptional responses, with a substantial number of DEGs uniquely enriched in Wk-1 and Wk-4 samples (Fig. 4A). The unsupervised hierarchical clustering of gene expression demonstrates a distinct profile associated with each phase of remodeling (Fig. 4B). Genes such as Postn (Periostin), Tgfb3 (TGF-beta 3), and Fn1 (fibronectin 1) were upregulated post-MI, with peak expression at Wk-1, indicating their active ECM remodeling and fibrotic signaling early after MI (Fig. 4C). Gene ontology (GO) enrichment analysis revealed significant pathways related to collagen biosynthesis, extracellular matrix assembly, and collagen fibril organization at Wk-1, highlighting the role of FBs in scar formation (Fig. 4D, top). However, most of the overexpressed genes in post-MI Wk-4 were enriched with smooth muscle cell differentiation, chondrocyte regulation, and tissue development (Fig. 4D, bottom), suggesting a functional and phenotypic shift toward tissue stabilization and structural remodeling. Key ECM-associated genes such as Col1a1, Col3a1, Col1a2, Col5a2, Col27a1, and Col14a1 were significantly upregulated post-MI, particularly at Wk-1 (Fig. 4E). The expression of Loxl3 and Serpinh1, which are involved in collagen cross-linking and stabilization, was also increased (Fig. 4E). At Wk-4, we observed increased expression of genes involved in smooth muscle-like fibroblast differentiation, including Cfh, C3, Comp, and Smad6. (Fig. 4F). It is more appropriate to refer to this as myofibroblast differentiation rather than smooth muscle cell differentiation, as the cluster originates from FBs and myofibroblasts also share a transcriptional profile similar to that of smooth muscle cells. Furthermore, transcriptional regulators involved in chondrogenic signaling such as, Zbtb16, Thrb, Sox6, and Tgfrb1, were also upregulated during Wk-4 (Fig. 4G). These results highlight a biphasic activation pattern in FBs, suggesting a temporal shift in FBs function from ECM deposition and fibrosis in the acute phase (Wk-1) to a more specialized differentiation, tissue remodeling and stiff ECM during the chronic phase (Wk-4), underscoring the dynamic role of FBs in post-MI repair. Additionally, we have looked at the expression of cardiogenic genes (Typically associated with CMs) in FBs to investigate the cluster heterogeneity within FBs. We observed an increase in some of these genes, including Mef2c, Gata4, Gata6 and Tbx20 during the Wk-1 and Wk-4 post-MI compared to the control (Fig. 4H). Further examination of the FBs subclusters using pseudo time analysis confirms the biphasic responses of FBs from ECM deposition and fibrosis at the early phase and differentiation, tissue remodeling and stiff ECM during the late phase (Fig. 4I-J).

### Activated FBs-to-CMs communication suggests pro-survival stress remodeling in CMs post-MI

**Fig. 5:**
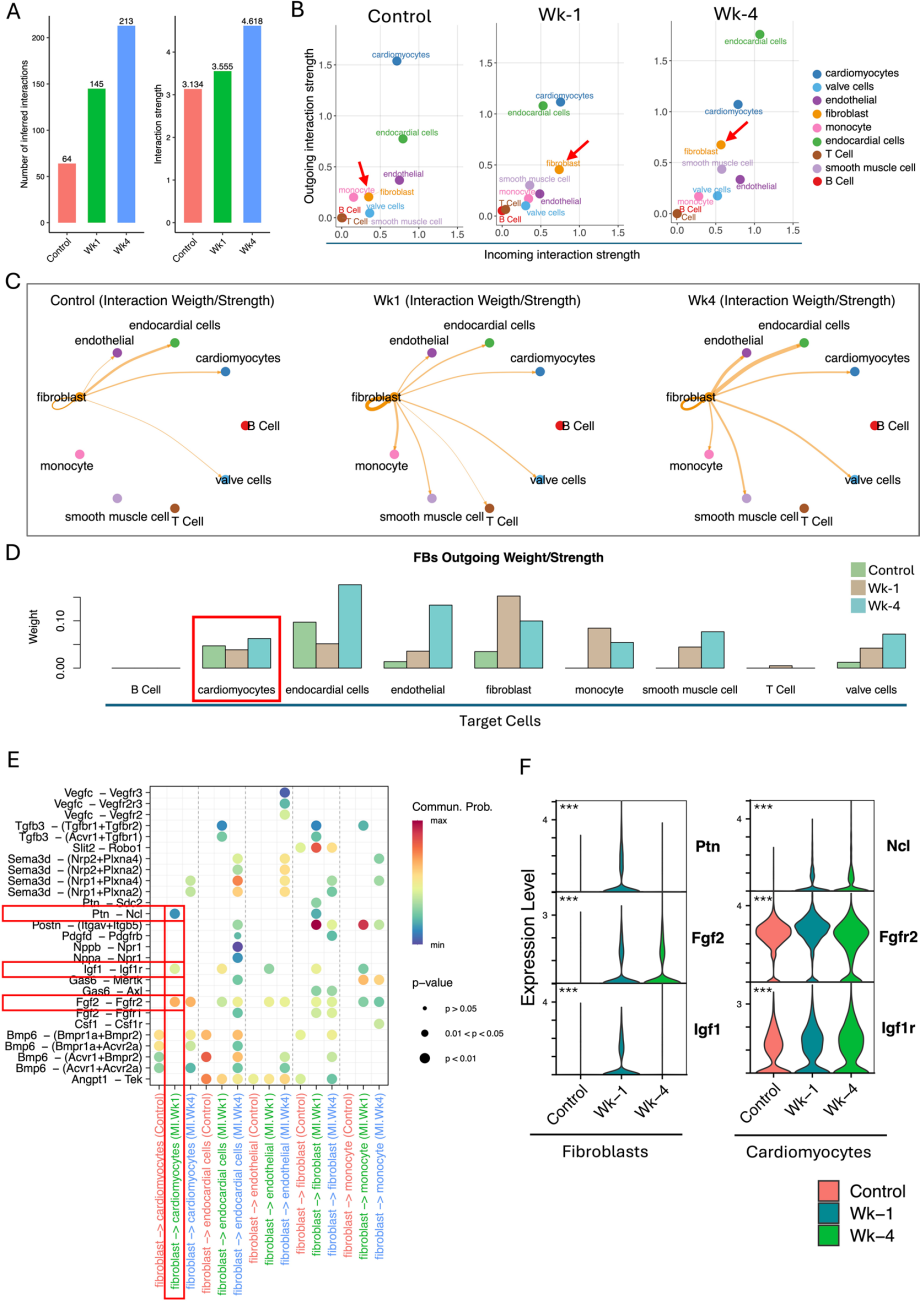
Differential secretory signaling between cardiac cells post-MI. **A** The total number of ligand-receptor interactions and their strength (as predicted by CellChat) for all cell types gradually increased in post-MI Wk-1 to Wk-4. **B** The scatter plot shows the change in outgoing and incoming interaction strength for different cell types in control, post-MI Wk-1 and Wk-4 samples. The outgoing strength of FBs was increased gradually during Wk-1 and Wk-4 post-MI as indicated by red arrow. **C** The interaction weight for the outgoing signaling in FBs demonstrates an overall increase in outgoing signals. **D** The bar plot showing the change in outgoing signaling weight/strength from FBs to all other cells. **E** The dot plot demonstrated outgoing signaling communications from FBs to other cell types that were significantly enriched in control, post-MI Wk-1 and Wk-4 samples. Highlighted with the red boxes are the three signaling pathways that are specifically activated during the post-MI Wk-1, demonstrating the communication between FBs and CMs. The signaling pathways from FBs to ECs that are highlighted during Wk-4 are highlighted with blue boxes. **F** Violin plot showing the expression change of the ligands (Ptn, Fgf2, and Igf1) in FBs and their corresponding receptors (Ncl, Fgfr2, and Igf1r) in CMs.

Beyond the intrinsic CMs response as described earlier, the progression of post-MI remodeling is critically governed by the dynamic intercellular communication within the injured myocardium. FBs adopt an activated phenotype and engage in extensive signaling with surviving CMs, influencing their function, survival, and hypertrophic responses. To elucidate the key molecular dialogues involved in this FB-CM crosstalk, we performed the cell-cell communication analysis using CellChat. We have looked at the ligand-receptor (LR) interactions and identified the differential signaling pathways during the post-MI stress response. The total number of interactions and their strength across all cell types were progressively increased from control to post-MI Wk-1 and Wk-4 (Fig. 5A). The outgoing signaling strength for FBs increased during post-MI Wk-1 and Wk-4, suggesting enhanced secretory signaling as a response to MI (Fig. 5B). The outgoing communication from FBs increased not only toward other cardiac cell types but also showed a marked rise in autocrine (self-) signaling, particularly during WK-1 post-MI (Fig. 5C). Further, to identify key signaling pathways mediating the interaction between FBs and CMs, we specifically looked at the LR pairs associated with FB-derived outgoing signaling. While the overall interaction strength from FBs to CMs remained largely unchanged, as indicated by the arrow width and the bar plots (Fig. 5C-D), the composition of active signaling pathways at Wk-1 post-MI differed substantially from both control and Wk-4 post-MI conditions (Fig. 5E). This analysis revealed several potential communication axes, with particularly strong evidence for the activation of Igf1-Igf1r, Ptn-Ncl, and Fgf2-Fgfr2 signaling from FBs towards CMs that are specifically activated during Wk-1 post-MI (Fig. 5E). The activation of these pathways suggests a FBs-driven effort to support CM survival and stress adaptation, potentially through anti-apoptotic signaling and increased glucose uptake. To support the predicted signaling interactions between FBs and CMs, we examined the expression patterns of ligands and their corresponding receptors (Fig. 5F). The results showed a notable upregulation of Ptn, Fgf2, and Igf1 specifically in FBs at WK-1 post-MI, consistent with their involvement in active signaling during this time point. Correspondingly, the receptors Ncl, Fgfr2, and Igf1r were expressed in CMs, confirming the potential for functional ligand-receptor interactions. The expression profiles of these signaling components further supports the hypothesis that FB-derived paracrine signaling may contribute to CMs survival and remodeling during the acute phase of MI. The dot plots for cell-specific key signaling pathways detected for CMs, FBs, ECs and endocardial cells (EDCs) are provided in the Supplementary Fig. 4.

### Fibroblast-mediated Sema3d and Vegfc signaling potentially implicates pro-angiogenic activity during late cardiac remodeling phase

Another notable secretory signaling reported from FBs is semaphorin 3 d (Sema3d), which was predominantly observed during the late phase of ischemic stress i.e. post-MI Wk-4, specifically from FBs to ECs and EDCs (Fig. 5D). While Sema3d has been reported in some contexts to be pro-angiogenic and promote vascular maturation and stability, primarily through signaling via Nrp1, it’s role specifically in FBs-derived signaling has not been established^37^. However, previous studies have implicated Sema3d signaling in vasculogenesis during heart development, suggesting a potential role in vascular repair and remodeling^38^. In our analysis, the upregulation of Sema3d along with the Vegfc signaling from FBs to ECs at Wk-4 post-MI suggests that FBs may contribute to pro-angiogenic signaling aimed at supporting vascular repair and stabilization during the late phase of cardiac remodeling. However, we do not report any major transcriptional changes in ECs population during Wk-1 and Wk-4 post-MI.

## Discussion

The complex pathophysiological response of the myocardium to ischemic injury involves a cascade of metabolic and structural changes, ultimately leading to CM death and irreversible tissue damage^19^. However, the endogenous regenerative capacity of the heart is limited, and therapeutic interventions to restore cardiac function remain a critical challenge^39,40^. Recent advancements in snRNA-seq have provided novel insights into the transcriptional profiles of various cardiac cell types following myocardial infarction^41^. These high-resolution analyses have shed light on the compositional, transcriptional, and functional alterations in CMs and other key cell populations, including FBs, ECs, and other resident cardiac cell types, during the pathological remodeling process^13,30,42–44^. The decrease in CMs, ECs, and EDCs at Wk-1 post-MI, contrasted with the proliferation of FBs and immune cells, highlights the immediate response to ischemic injury. The restoration of cellular proportions by Wk-4 underscores the adaptive response in the heart. These compositional shifts, coupled with the emergence of specific clusters within CMs, FBs, and ECs, point to a dynamic cellular landscape orchestrating repair and remodeling^4,45^. This study highlights significant transcriptional changes in CMs following myocardial infarction (MI), demonstrating a clear shift from a non-stressed state (CM-1) in control hearts to a stressed phenotype (CM-2 and CM-3) in post-MI conditions. These findings provide insight into the molecular mechanisms underlying CMs dysfunction, metabolic remodeling, and structural adaptation during the post-MI progression. In this study, we have used a single biological replicate for each sample; however, we used the external dataset published by other researchers in GEO with more biological replicates to confirm the similar expression pattern of the genes reported in this study (Supplementary Fig. 5).

A key observation is the loss of fatty acid beta-oxidation enrichment during Wk-1 post-MI, indicating a shift in CM metabolism after MI. This aligns with previous reports that ischemic stress forces CMs to increase glucose utilization at the expense of fatty acid oxidation^46–49^. The observed downregulation of mitochondrial complex proteins and oxidative phosphorylation (OxPhos) pathways at Wk-1 further supports this metabolic switch. However, the partial recovery of OxPhos pathways at Wk-4 suggests an adaptive response, where the mitochondrial function is restored as the heart stabilizes^50^. This metabolic plasticity may be an adaptive response aimed at optimizing energy efficiency in the ischemic heart, but prolonged dysregulation could contribute to contractile dysfunction^51^. The upregulation of hypertrophic markers Myh7 and Nppb at both Wk-1 and Wk-4, coupled with the downregulation of Myh6, indicates that surviving CMs undergo hypertrophy to compensate for lost contractile function. This hypertrophic response, while initially protective, may contribute to long-term pathological remodeling and heart failure progression. The significant downregulation of Mylk4, a key regulator of myosin light chain phosphorylation, suggests impaired contractility in post-MI CMs^52,53^. The concurrent upregulation of Ankrd1, a gene involved in sarcomere assembly and mechano-sensing, further indicates structural stress and maladaptive remodeling^53–55^. However, a further increase in the expression of Ankrd1 in CMs at WK-4 suggests that CMs remain in a pathological state, potentially contributing to ongoing contractile dysfunction in the infarcted heart^56,57^. The observed changes highlight the complexity of CM adaptation post-MI, balancing energy metabolism shifts, and contractile dysfunction. While the partial recovery of mitochondrial metabolism suggests compensatory response, the persistent hypertrophy and fibrosis-related changes indicate ongoing stress in the surviving CMs. Future therapeutic strategies should consider targeting metabolic pathways to enhance mitochondrial function, mitigating excessive fibrosis, and improving contractile recovery to prevent progression of long-term heart failure.

The observed transcriptional changes in FBs post-MI highlight their essential role in ECM remodeling and fibrosis. The early upregulation of collagen-related genes (Col1a1, Col3a1, Col5a2) at Wk-1 suggests an acute fibrotic response, necessary for stabilizing the infarcted tissue. The expression patterns of Postn and Tgfb3 align with previous studies that identify these factors as key drivers of FB activation and myofibroblast differentiation post-MI^58^. TGF-beta signaling, particularly through Tgfb3, is a major regulator of fibrosis, and its upregulation at Wk-1 suggests a pro-fibrotic environment that may transition into a remodeling phase by Wk-4. Interestingly, the decline in FB-associated ECM genes by Wk-4 suggests a resolution phase where fibrosis stabilizes. The increased expression of Fn1 at Wk-1, followed by a decrease at Wk-4, further suggests dynamic remodeling in the infarcted heart as endogenous cardiac repair The GO enrichment analysis and corresponding gene expression data provide important insights into the dynamic roles of FBs during post-MI remodeling. At Wk-1 post-MI, FBs exhibited strong enrichment for biological processes related to collagen fibril organization, ECM assembly, and cell-substrate junction assembly, consistent with their well-established role in fibrotic scar formation and tissue stabilization in the early phase of injury. In Wk-4, the GO terms enriched in FBs shifted toward myofibroblast differentiation, chondrogenic regulation, and tissue morphogenesis, suggesting a transition from matrix deposition to a more specialized, differentiation-oriented phenotype. The increased expression of ECM proteins at Wk-1 post-MI followed by the overexpression of bone/cartilage genes at Wk-4 has previously been reported in FBs^59^. Further our cell-cell communication analysis reveals increased Tgfb3 and Postn signaling at Wk-1 and Bmp6 signaling at Wk-4 post-MI in FBs. This biphasic transcriptional pattern highlights how FBs initially contribute to ECM deposition and wound closure and subsequently adopt diverse phenotypes that may support vascular maturation, immune modulation, or mechanical reinforcement of the healing myocardium. These findings underscore the temporal complexity of FB function post-MI and suggest that targeting specific FB states or transitions may offer therapeutic opportunities to balance scar formation with functional tissue regeneration. Additionally, the cardiomyocyte associated genes expressed in fibroblasts may play an important role in facilitating the survival process after MI^60^.

Following the characterization of CMs and FBs specific transcriptomic alterations post-MI, we extended our investigation to explore the crucial intercellular communication between FBs and CMs. Given the well-established role of FB-CM crosstalk in post-infarction remodeling, we used cell-cell communication analysis to elucidate the key molecular dialogues involved in this FB-CM crosstalk. Our findings suggest an upregulation of critical growth factor signaling pathways, including Igf-1/Ifg1r, Ptn/Ncl, and Fgf2/Fgfr2, within CMs following myocardial infarction. The activation of these pathways is highly significant to cellular survival and metabolic adaptation during ischemic stress. Igf1/Ifg1r signaling is a potent pro-survival pathway in CMs^61^. Its activation, often via PI3K/Akt and MAPK pathways, helps counteract apoptotic signals triggered by ischemia/reperfusion injury^62^. This is crucial in the acute phase to salvage viable myocardium. IGF-1 signaling, like insulin signaling, promotes glucose uptake (e.g., via Glut4 translocation) and glycolysis^63^. This can be beneficial particularly when oxidative phosphorylation might be impaired due to hypoxia which was significantly suppressed during post-MI Wk-1. While the enriched pathways we identified are consistent with previously reported pro-survival and metabolic adaptation mechanisms in CMs post-MI, confirming these relationships will require functional assays or multimodal validation Similarly, Fgf2/Fgfr2 signaling plays a critical role in cardio-protection and is a powerful inducer of angiogenesis in the ischemic heart^64,65^. Additionally, Fgf2 signaling can activate intracellular pathways that directly preserve mitochondrial integrity and reduce oxidative damage^66,67^. Ptn/Ncl signaling, although not studied in cardiac ischemic stress, studies suggest its role in heart development and promoting angiogenesis^68,69^. Further, the cell communication from FBs to ECs suggests that FBs may adopt a pro-angiogenic role during the late phase of cardiac remodeling through Sema3d and Vegfc signaling, potentially contributing to vascular stabilization. These signaling pathways could be of novel interest for studying cardiac repair after ischemic stress. While our cell–cell communication analysis highlights potential signaling pathways involved in post-MI remodeling, these findings are based on predictive ligand–receptor modeling and lack direct experimental validation. Therefore, interpretations such as “pro-survival” or “stress-adaptive” signaling observed here should be considered hypothesis-generating rather than conclusive, as it requires functional confirmation through protein-level or perturbation-based studies.

This study provides a comprehensive transcriptomic analysis of the post-MI heart, revealing cell-type-specific transcriptional shifts and intercellular signaling changes that correlate with the processes of cardiac remodeling. The observed CMs transcriptional changes, characterized by metabolic shifts from fatty acid oxidation to glycolysis, and hypertrophic responses underscore the complexity of myocardial adaptation to ischemic stress. Furthermore, FBs demonstrated temporally regulated transcriptional programs during post-MI remodeling, transitioning from a fibrotic, matrix-producing phenotype in the early phase to a more specialized, differentiation-associated state in the later phase. This phenotypic plasticity underscores the multifaceted roles of fibroblasts, including scar formation, support of vascular recovery, and enhanced expression of cardiomyocyte-associated genes that becomes more prominent during the later phase. The cell-cell communication analysis further highlights the evolving interplay between FBs, and CMs, particularly the activation of Igf1-Igf1r, and Fgf2-Fgfr2 suggesting the FBs-driven survival and stress adaptation of CMs. Together, these findings emphasize the need for therapeutic strategies aimed at modulating metabolic pathways, limiting excessive fibrosis, and enhancing vascular recovery to improve post-MI cardiac function and long-term outcomes. Future studies should focus on targeting key molecular regulators identified in this study to develop interventions that can optimize cardiac repair while preventing adverse remodeling and heart failure progression.

### Limitations

While this study provides detailed insights into the transcriptional and signaling changes occurring during the Wk-1 and Wk-4 post-MI, it does not include earlier time points immediately following infarction, when extensive cardiomyocyte death and acute inflammation dominate. These early stages are known to exhibit high transcriptional heterogeneity and intense intercellular communication among immune, endothelial, and stromal cells, which likely shape the downstream remodeling responses observed at Wk-1 and Wk-4. Consequently, our analysis primarily reflects the later adaptive phases of remodeling. Future studies incorporating earlier post-injury intervals will be essential to capture the full temporal trajectory of molecular and cellular events driving myocardial repair. Furthermore, while snRNA-seq provides valuable insights into the transcriptional landscape of the infarcted heart, several methodological constraints are important to consider. The nuclear transcriptome represents only a subset of total cellular RNA and is biased toward un-spliced or nascent transcripts, which can reduce detection sensitivity compared to whole-cell RNA-seq. Consequently, low-abundance or cytoplasmic mRNAs, particularly those encoding metabolic or contractile proteins, may be underrepresented, leading to dropout effects and limited detection of subtle transcriptional shifts. Additionally, nuclear RNA abundance does not always correlate with cytoplasmic mRNA or protein levels due to post-transcriptional and translational regulation. Despite these technical limitations, snRNA-seq remains highly advantageous for profiling preserved nuclei from fibrotic or necrotic myocardial tissue, where intact cells are difficult to isolate, allowing robust characterization of cell-type-specific responses during cardiac remodeling.

Furthermore, this study utilized a single biological replicate per condition, which limits the ability to capture inter-animal variability and to make broad population-level inferences. To partially mitigate this constraint, we focused on robust, cell-type-resolved transcriptional signals reproducible across large numbers of nuclei and applied conservative differential-expression thresholds. Major observations, including cardiomyocyte metabolic alterations, fibroblast activation states, and key ligand–receptor interactions, were further supported by concordant patterns in independent publicly available datasets. Nevertheless, future studies incorporating larger biological cohorts will be necessary to fully validate and extend these findings.

## Methods

### Ethical statement

All the animal procedures were performed in accordance with ARRIVE guidelines 2.0 and with the approval of the Institutional Animal Care and Use Committee of The Ohio State University and conformed to the Guide for the Care and Use of Laboratory Animals, published by the National Institutes of Health^70^.

### Induction of myocardial Infarction

Myocardial infarction (MI) was induced under anaesthesia (2% isoflurane) in 8–9-week-old male C57BL/6J wild-type mice (The Jackson Laboratory, USA) by permanent ligation of the left anterior descending (LAD) coronary artery, as previously described^71–73^. Briefly, the chest cavity was opened with a small incision at the third or fourth intercostal space. The heart was exposed, and the LAD was identified and permanently ligated 1–2 mm below the left auricle using a 7–0 polypropylene suture. Successful occlusion was confirmed by pallor of the anterior left ventricular wall. The chest cavity was closed with 7–0 vicryl sutures, and the skin was sutured with 6–0 polypropylene sutures.

### Assessment of cardiac function via ECG and M-mode echocardiography

Mice were anaesthetized using isoflurane (1.5%) and were placed in a supine position on a heated surgical table. The mice were connected to three electrodes: one on the left foreleg, one on the right foreleg, and one on the right hind leg. The ECG signals were recorded on a PowerLabSupport software using the ECG Analysis Module (ADInstruments, USA). The readings were taken before the surgery, 5–10 min after the surgery, and on the Wk-1 and Wk-4 after the surgery. RR interval, P wave duration, PR interval, QRS, JT and QT durations were recorded. Further, to assess the cardiac function via echocardiography the mice were anaesthetized as described before and transthoracic M-mode echocardiography measurements were conducted at baseline and at Wk-1 & Wk-4 post-MI using a Vivo F2 ultrasound imaging system (Fujifilm visualsonics, USA). Heart size and shape were calculated using the M-mode and two-dimensional long-axis image plane of the LV. Measurements were averaged from 5 cardiac cycles. The data were used to estimate the percentage LV ejection fraction (EF) & fractional shortening (FS), Left ventricular internal dimension at end-systole (LVIDs), Left ventricular internal dimension at end-diastole (LVIDd), Cardiac output (CO), Stroke volume (SV), End systolic volume (ESV) and End diastolic volume (ESV).

### Assessment of cardiac fibrosis via Masson’s Trichrome (MT) staining

Masson-trichrome immunohistochemical staining was performed to assess the extent of cardiac fibrosis in all the groups (Control, WK-1 and WK-4 post MI; *n* = 3/group). After measurement of hemodynamic function, the animals were deeply anesthetized with 5% isoflurane and when no longer responsive to a firm toe pinch, the chest is rapidly opened and the great vessels were cut to remove the heart, resulting in exsanguination and euthanasia of the animals. Isolated hearts were then washed with ice-cold phosphate buffered saline and fixed in 4% paraformaldehyde. Paraformaldehyde-fixed samples were embedded in OCT and cut into 6μm sections for MT staining for detection of fibrosis extension. The tissue slides were visualized using EVOS M7000. 

### Single-nuclei RNA sequencing, data processing and quality control

The mice were euthanized as discussed above, and the atria were removed from the mice hearts immediately after harvesting, and only the left ventricular tissue was isolated from a single biological replicate of control and post-MI (Wk-1 & Wk-4) and used for nuclei isolation, library preparation, and sequencing. The single-nucleus suspension was processed using the 10x Genomics Chromium platform for droplet-based encapsulation and barcoding. Library preparation was performed using the Chromium Single Cell 3’ Reagent Kit (Next GEM v3.1). The generated libraries were sequenced with ~ 200 million PE150 reads per sample on Illumina NovaSeq. The sequencing reads were analyzed with the mouse reference genome mm10 using Cell Ranger v7.1.0 (Singulomics, USA).

Low-quality nuclei, defined as those with < 500 and > 5000 number of detected genes or > 10% mitochondrial transcripts, were filtered out to remove ambient RNA contamination using Seurat (v5.0.2)^74^. The total nuclei after filtering were 5884 for Control, 5946 for Wk-1, and 4212 for Wk-4. The average number of UMI counts obtained for Control, Wk-1 and Wk-4 samples were 2742.5, 3078.8, and 3902.8, respectively. Doublets were identified and removed using scDbFinder (v1.18.0), and batch effects were corrected using Harmony (v1.2.3)^75,76^.

### Clustering, cell annotation and differential gene expression analysis

Following quality control, normalized gene expression data were processed using the Seurat pipeline. Feature selection was performed to identify highly variable genes, and principal component analysis (PCA) was conducted to reduce dimensionality. The top 20 principal components (PCs) were selected based on ElbowPlot, followed by Uniform Manifold Approximation and Projection (UMAP) for visualization of nuclei clustering. Unsupervised clustering was performed using Shared Nearest Neighbor (SNN) modularity optimization, with a resolution of 0.1–0.9, to identify distinct cellular populations. Cluster resolution of 0.9 was used to fully resolve the subclusters. The cell-type annotation was performed using the Tabula Muris Senis dataset as a reference. The TabulaMurisenisData package in R was used to assign preliminary cell identities based on transcriptomic profiles^77^. To further refine annotations, canonical marker gene expression was examined across clusters, ensuring accurate classification of cells. Any ambiguous clusters were manually inspected and reassigned based on differential gene expression and known cardiac cell markers. Differential gene expression analysis was performed using the FindMarkers function in Seurat with a Wilcoxon rank-sum test, applying an adjusted p-value threshold of < 0.05. Pseudotime analysis on the FB cell cluster was performed using the monocle3 (v1.4.26) package in R.

### Cell–cell communication analysis

Cell–cell communication was analyzed using the CellChat R package (v2.1.2), which allows for the identification of signaling pathways between cellular populations based on their gene expression^78^. The normalized and clustered gene expression matrix from *Seurat* was converted into a *CellChat* object using *createCellChat* function, which was used as input for downstream analysis. Gene expression profiles were mapped to signaling ligand-receptor pairs derived from *CellChatDB* databases. Ligand-receptor interactions for each cell type within the dataset were modelled and the probability of communication between clusters based on the expression of ligands, receptors, and signaling pathway activity were computed using the *computeCommunProb* function. The communications were filtered for a minimum 30 cells per group using the ‘*min.cells’* parameter. The resulting communication networks were visualized using various CellChat-specific plotting functions.

### Functional annotation and gene set enrichment analysis

Functional annotation of the genes for gene ontology (GO) terms was performed using the enrichGO function of clusterProfiler package (v4.12.6) in R^79^. The enrichment plot were generated using enrichplot (v1.24.4) package^80^. The geneset enrichment analysis (GSEA) of differentially expressed genes was performed using GSEA software (v4.3.3)^81^.

### Statistical analysis

All the statistical analyses for snRNA-seq data were performed using R and Python-based bioinformatics pipelines. The quality control, normalization, and clustering were conducted using Seurat. Batch effects across samples and tissues was corrected using Harmony (v1.2.3), and differentially expressed genes (DEGs) were identified using the Wilcoxon rank-sum test. P-values were adjusted using the Bonferroni correction method. Gene expression differences for selected genes between the sample groups were assessed using the Kruskal-Wallis test, and the resulting p-values were adjusted for multiple comparisons using the Benjamini-Hochberg (BH) method to control the false discovery rate (FDR). An adjusted p-value (FDR) of < 0.05 was considered to indicate statistical significance. For functional characterization of MI, all tests were performed using one-way ANOVA followed by Tukey’s post hoc test for multiple comparisons on Graphpad Prism software (v10). All statistical tests were two-tailed, and p-values < 0.05 was considered statistically significant.

## Supplementary Information

Below is the link to the electronic supplementary material.

Supplementary Material 1

Supplementary Material 2

## Data Availability

The raw and processed files for snRNASeq data were made available via NCBI GEO database using the accession number **GSE302892**. The reviewer’s access code for snRNASeq data is **shgloyksjlyznif**. The other data used in this study can be made available upon considerable request.

## Abbreviations

ANOVA: Analysis of Variance
CMs: Cardiomyocytes
CO: Cardiac output
DEGs: Differentially Expressed Genes
ECG: Electrocardiography
Echo: Echocardiography
ECs: Endothelial Cells
EDCs: Endocardial Cells
EDV: End diastolic volume
EF: Ejection Fraction
ESV: End systolic volume
FBs: Cardiac Fibroblasts
FS: Fractional Shortening
GO: Gene Ontology
GSEA: Geneset Enrichment Analysis
LAD: Left Anterior Descending artery
LV: Left Ventricle
LVIDd: Left ventricular internal dimension at end-diastole
LVIDs: Left ventricular internal dimension at end-systole
MI: Myocardial Infarction
MSE: Mean Squared Error
MT: Masson’s Trichrome
OxPhos: Oxidative Phosphorylation
PCA: Principal Component Analysis
PCs: Principal Components
scRNA-seq: Single-cellRNA sequencing
SNN: Shared Nearest Neighbor
snRNA-seq: Single-nuclei RNA sequencing
SV: Stroke volume
UMAP: Uniform Manifold Approximation and Projection
Wk-1: Week 1
Wk-4: Week 4
