# Supplementary material for: Transcriptional remodeling of cardiomyocytes and fibroblasts during post-myocardial infarction recovery
Source: Sci Rep. 2026 Mar 4;16:12120. doi: 10.1038/s41598-026-41631-y (PMC13076631; doi:10.1038/s41598-026-41631-y)
Supplement: Supplementary file 1 — Supplementary Material 1 [file 41598_2026_41631_MOESM1_ESM.docx]

**Supplementary Material-1 (Supplementary Figures)**

**Supplementary Fig. 1**


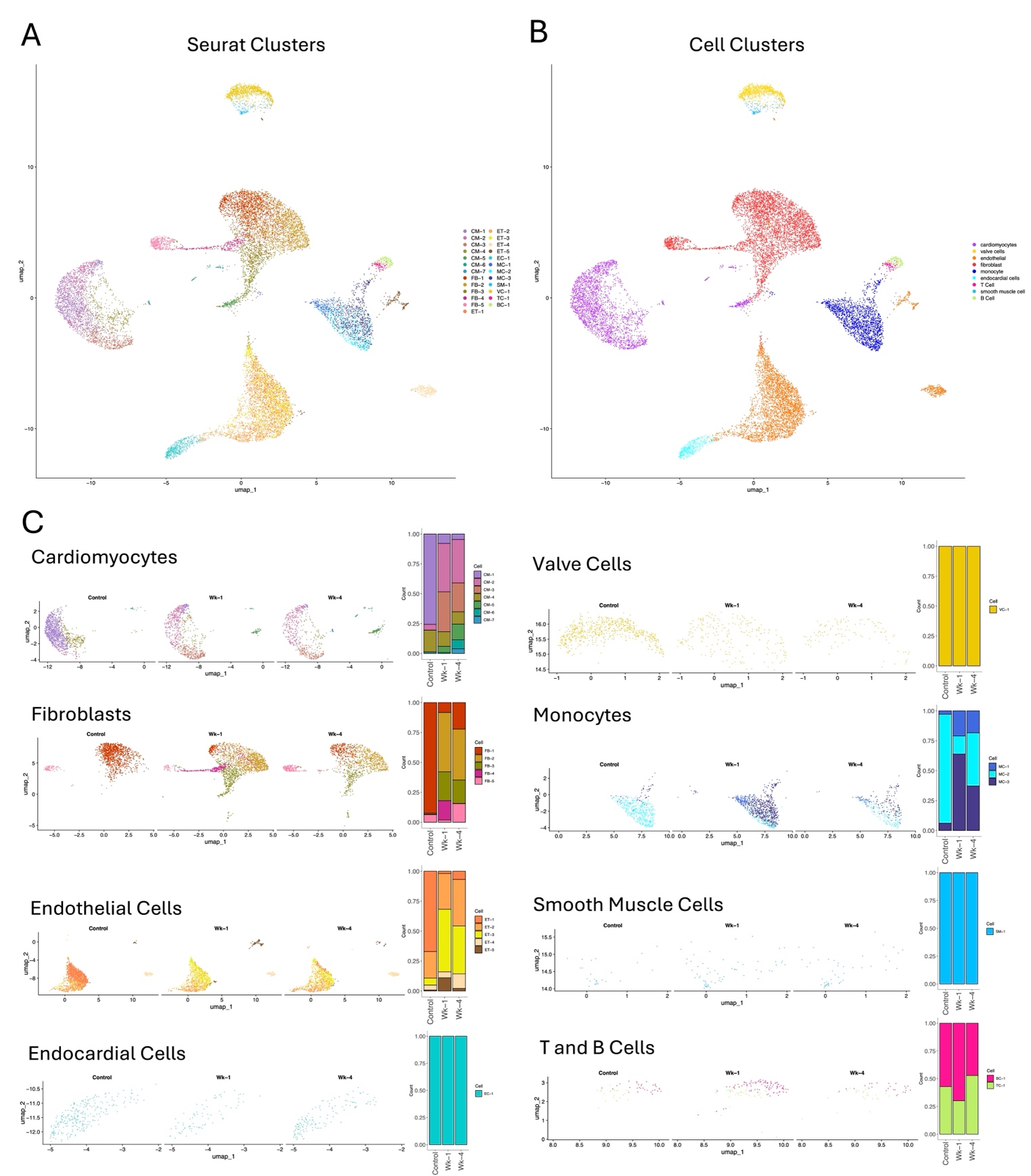


**Supplementary Figure 1: Cluster Heterogeneity: A)** UMAP representation demonstrating total 25 clusters that were identified after clustering analysis and **B)** nine annotated cell clusters. **C)** The distribution of clusters across samples for each cell type showing the change the cluster heterogeneity during post-MI.

**Supplementary Fig. 2**


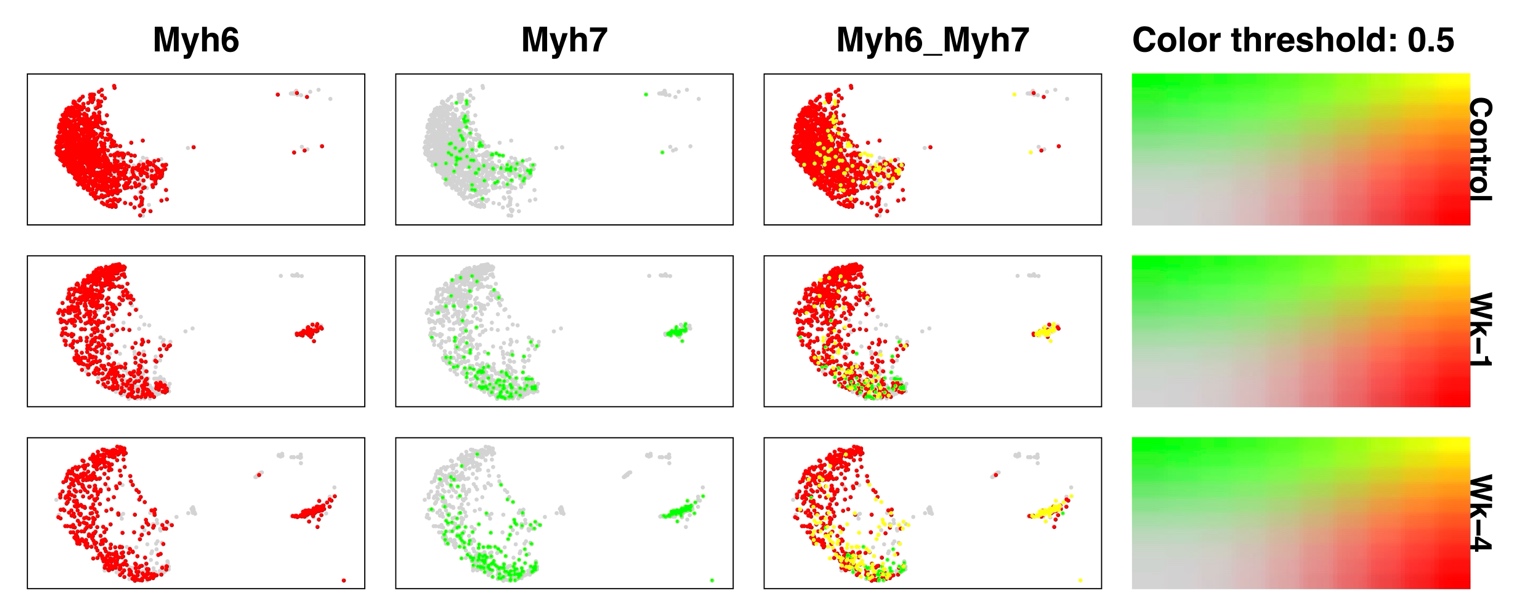


**Supplementary Figure 2: Feature plot of Myh6 & 7.** The feature plot showing the expression of Myh6 (Red) and Myh7 (Green) in CM cluster. In the merged panel more green cells are observed in Wk-1 and Wk-4, suggesting increased expression of Myh7 in a subpopulation of CMs.

**Supplementary Fig. 3**


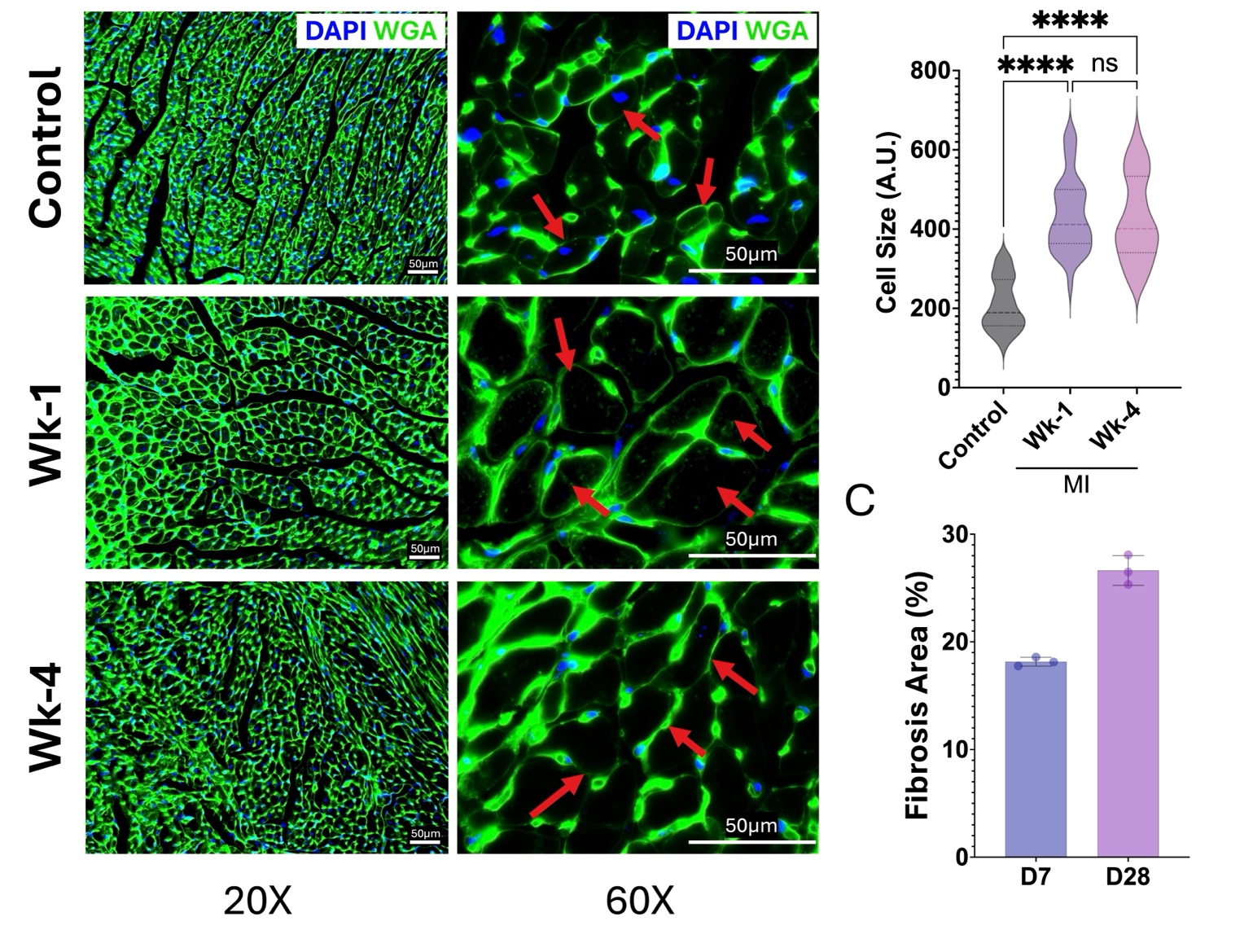


**Supplementary Figure 3: WGA staining. A)** WGA staining of heart section showing the increased cell size (at 20X and 60X) post-MI Wk-1 and Wk-4 as indicated by arrows. **B)** Individual cardiomyocyte size was assessed using ImageJ (National Institutes of Health). Mean cardiomyocyte cross-section area (um2) in peri-infarct region was evaluated by measuring 50 cells per heart. n = 3 per group; values are shown as mean +_SEM. **C)** The quantification of area of infarction measured from MT staining, n=3 per group. ****p<0.0001; Blue, DAPI (nuclei); Green, Alexa Fluor 488 (WGA)

**Supplementary Fig. 4**

**
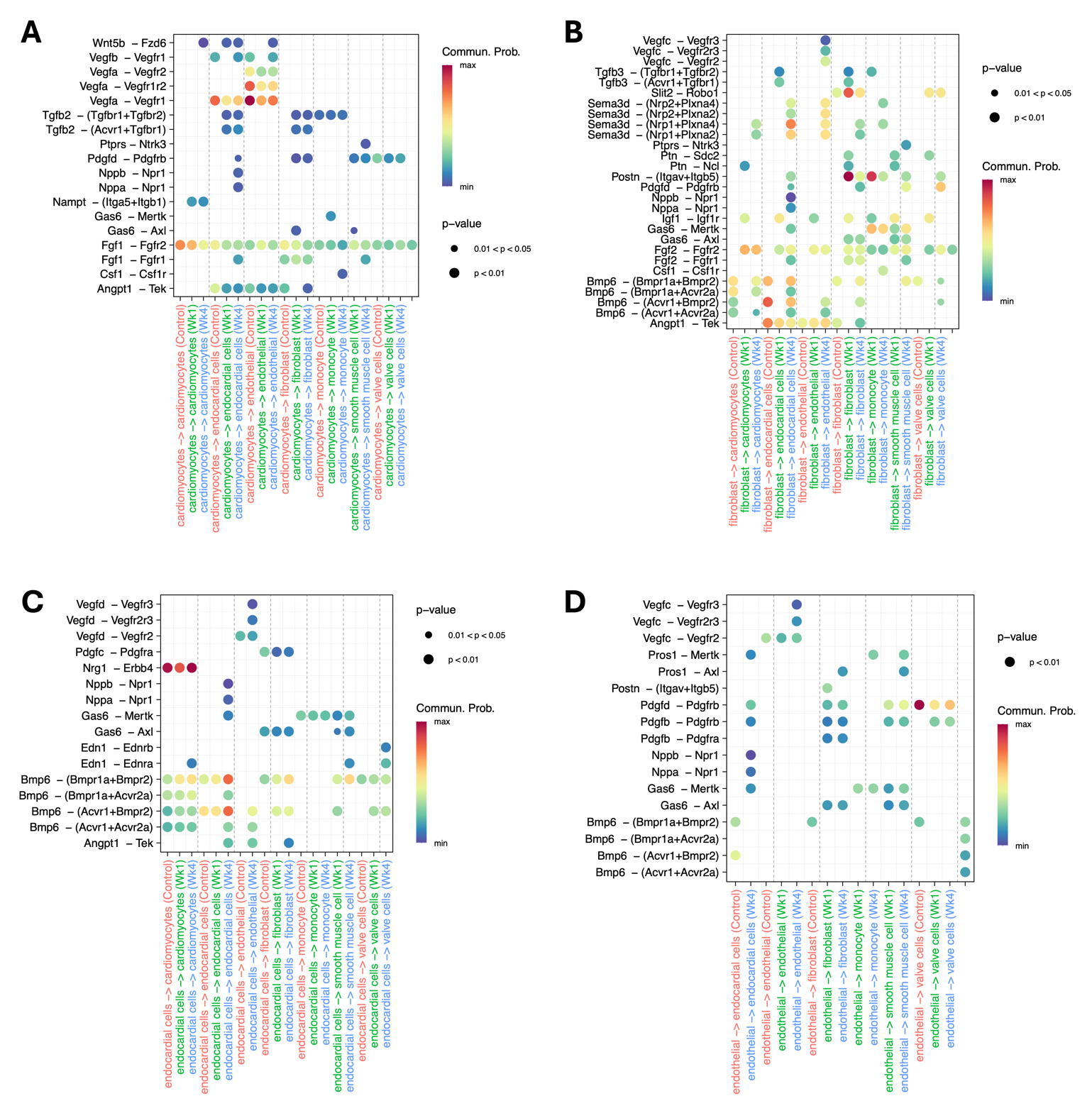
**

**Supplementary Figure 4: Alteration of signaling pathways involved in inter-cell communications.** The figure demonstrates the change in outgoing signaling for four cell types: **A)** Cardiomyocytes, **B)** Fibroblasts, **C)** Endothelial cells, and **D)** Endocardial cells. The singling probability is calculated for each of these four cells to all other cells across samples.


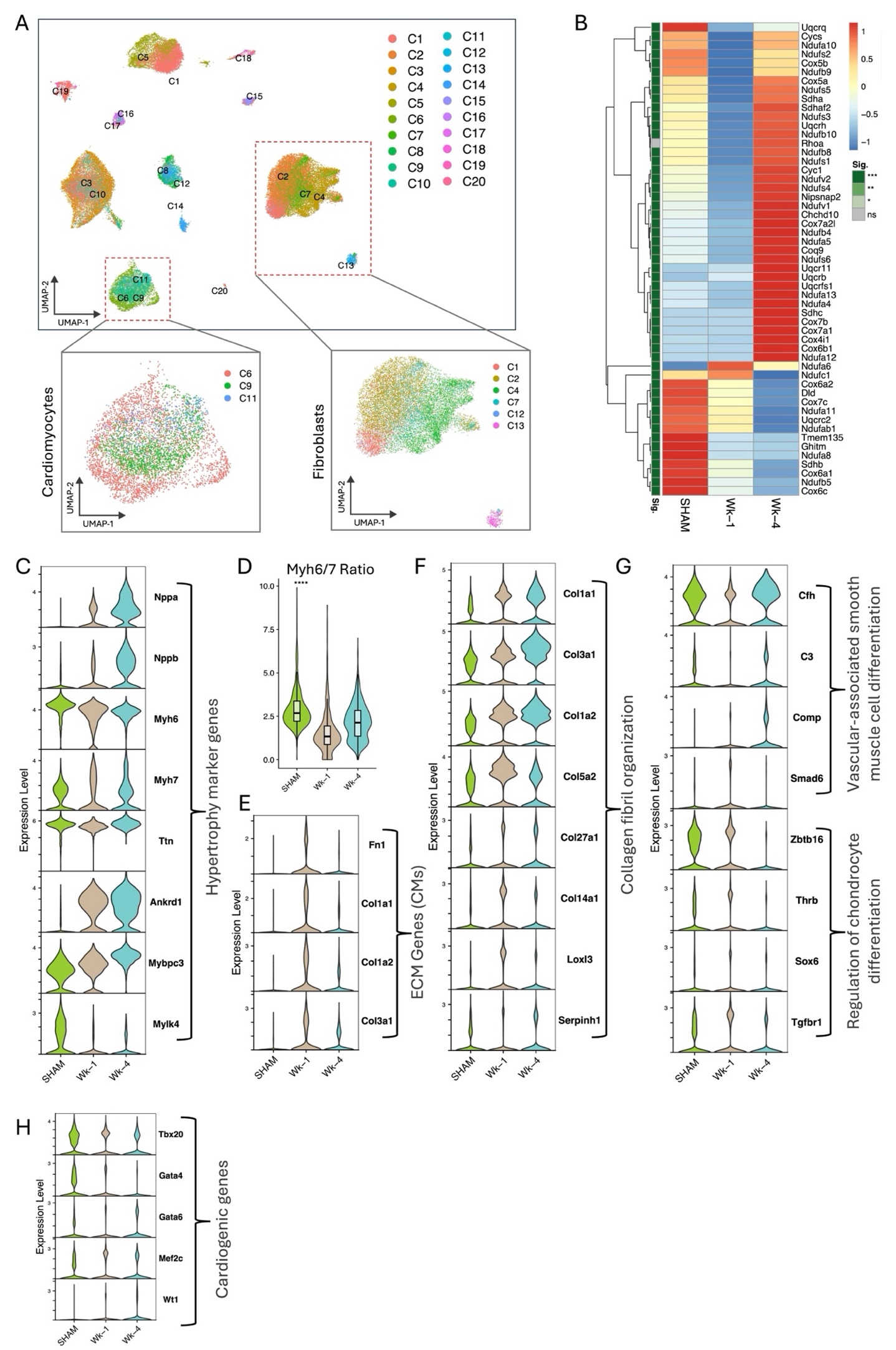


**Supplementary Figure 5**: **snRNA Seq analysis of external datasets.** **A)** UMAP demonstrating the cardiomyocytes and fibroblast clusters. B) Heatmap showing the change in Oxphos genes in cardiomyocytes. **C)** Violin plot showing the expression of hypertrophic genes. **D)** Myh6/7 expression ratio in CMs. **E)** Expression of ECM genes in CMs. **F-G)** The expression of collagen fibril organization (Col1a1, Col3a1, Col1a2, Col5a2, Col27a1, Col14a1), ECM regulation (Loxl3, Serpinh1), vascular-associated smooth muscle cell differentiation (Cfh, C3, Comp, Smad6), and genes involved in regulation of chondrocyte differentiation (Zbtb16, Thrb, Sox6, Tgfrb1) in FBs.
