# Supplementary material for: Transcriptional remodeling of cardiomyocytes and fibroblasts during post-myocardial infarction recovery
Source: Sci Rep. 2026 Mar 4;16:12120. doi: 10.1038/s41598-026-41631-y (PMC13076631; doi:10.1038/s41598-026-41631-y)
Supplement: Supplementary file 2 — Supplementary Material 2 [file 41598_2026_41631_MOESM2_ESM.docx]

**Supplementary Material-2 (Methodology)**

1. **Methodology for Wheat germ agglutinin (WGA) staining.**

To measure the cardiomyocyte area, mouse heart sections were stained with WGA (Alexa Fluor 488, Invitrogen, USA) and DAPI (Invitrogen, USA). Fluorescent signals were observed and recorded at 20X and 60X using EVOS M7000 (Invitrogen, USA). 10 fields were chosen at random for measuring cardiomyocyte cross-section area (CSA) at 20X through Celleste Image Analysis Software.

1. **Methodology for snRNA-seq Validation Data Processing and Analysis**

**Data Acquisition and Initial Processing**

Raw count matrices from publicly available datasets (GSE214611 for D0 and Wk1 samples, and GSE193290 for the Wk4 sample) were imported into the R environment. Data from individual samples were loaded using the Read10X function and converted into independent Seurat objects using the CreateSeuratObject function. Samples were assigned project identifiers ("SHAM", "Wk1", or "Wk4") for subsequent metadata tracking.

**Quality Control and Filtering**

Quality control (QC) metrics were calculated for each dataset. The percentage of mitochondrial genes was determined using the PercentageFeatureSet function with a "^mt-" pattern to identify mouse mitochondrial transcripts. VlnPlots were generated to visualize key metrics: the number of unique features (nFeature_RNA), the total number of counts per cell (nCount_RNA), and the percentage of mitochondrial reads (percent.mt).

Cells were filtered based on these metrics to remove low-quality cells and potential doublets. The following thresholds were applied uniformly across all samples:

- nCount_RNA < 20,000
- nFeature_RNA > 500 and < 4,000
- percent.mt < 10%

These thresholds ensure the retention of high-quality, viable nuclei for downstream analysis.

**Doublet Detection and Removal**

Putative doublets (two or more nuclei mistakenly captured as a single event) were predicted and removed from each filtered dataset. This was achieved using the scDblFinder package, through custom functions findDoublets and subsetting the data to retain only cells classified as "singlet" by the algorithm.

**Data Integration and Normalization**

Following QC and doublet removal, all individual sample objects were merged into a single integrated Seurat object using the merge function. Distinct cell IDs were added to identify the origin of each cell. The merged data underwent standard normalization using the "LogNormalize" method with a scale factor of 10,000. Subsequently, the top 2,000 most variable features were identified using the "vst" selection method via the FindVariableFeatures function. The data was scaled using the ScaleData function, and principal component analysis (PCA) was performed to reduce dimensionality using the RunPCA function.

To address potential batch effects arising from combining datasets generated at different times or from different sources, we employed batch correction using Harmony. The RunHarmony function was utilized, specifying 'orig.ident' as the variable for grouping samples during integration to align the data effectively.

**Clustering and Dimensionality Reduction**

Uniform Manifold Approximation and Projection (UMAP) was used for non-linear dimensionality reduction and visualization of the data. Initial UMAP visualization before and after Harmony integration confirmed the effectiveness of batch correction. Cell clustering was performed on the Harmony-corrected embeddings. The FindNeighbors function identified cell neighborhoods based on the top 30 dimensions, followed by the FindClusters function using a range of resolutions (0.1 to 1). A resolution of 0.3 was selected for final cluster assignment, which was then assigned to the 'seurat_clusters' metadata field.

**Cell Type Annotation**

Following data integration and clustering, cell clusters for “ventricular cardiomyocytes” and “fibroblasts” were annotated based on the expression of known, canonical marker. To quantify the expression of these markers within each cell, the AddModuleScore function from the Seurat package was used. This function calculates a cumulative expression score (module score) for each cell, representing the average expression of the genes in each module while controlling for background noise.
